# A CRISPR-Cas12a-Based Diagnostic Method for Japanese Encephalitis Virus Genotypes I, III, and V

**DOI:** 10.3390/bios13080769

**Published:** 2023-07-28

**Authors:** Namki Kwak, Bum Ju Park, Yoon-Jae Song

**Affiliations:** Department of Life Science, Gachon University, 1342, Seongnam-si 13120, Republic of Korea; namki0301@naver.com (N.K.); catagory95@naver.com (B.J.P.)

**Keywords:** Japanese encephalitis virus, diagnosis, CRISPR-Cas12a, DETECTR

## Abstract

The Japanese encephalitis virus (JEV) is prevalent in Asian countries, including Korea, Japan, China, Vietnam, and India. JEV is transmitted to humans by Culex mosquitoes. Despite extensive research efforts, no approved antiviral agents are currently available, although JE can be prevented by vaccination. DNA endonuclease-targeted CRISPR trans reporter (DETECTR) is a newly emerging CRISPR-Cas12a-based molecular diagnostic method combined with isothermal nucleic acid amplification. In this study, DETECTR with reverse transcription–recombinase polymerase amplification (RT-RPA) was effectively utilized for JEV diagnosis and detected down to 10 RNA copies for JEV genotype I (GI) and 1 × 10^2^ copies for both GIII and GV, achieving similar sensitivity to RT-PCR while displaying no cross-reaction with other viruses. A one-tube, one-temperature format of DETECTR was further developed, and its efficiency compared with that of conventional DETECTR.

## 1. Introduction

The Japanese encephalitis virus (JEV) is the underlying cause of Japanese encephalitis (JE) transmitted to humans by *Culex* mosquitoes. Pigs and birds serve as major reservoirs, and the virus is transmitted from infected animals to other hosts by mosquitoes. In addition, JEV-infected pigs can directly transmit the virus to uninfected pigs, and viral amplification is activated in infected pigs [1]. However, no person-to-person transmission has been reported to date, and humans are only infected when bitten by carrier mosquitoes. The *Culex tritaeniorhynchus* native to India, East Asia, and Southeast Asia is a major vector of JEV [2].

JEV belongs to the *Flaviviridae* family and has a 11 kb genome of positive single-stranded RNA encoding three structural proteins (capsid (C), pre-membrane (prM), and envelope (E)) and seven non-structural proteins (NS1, NS2A, NS2B, NS3, NS4A, NS4B, and NS5) [3,4]. The capsid protein forms a nucleocapsid. The pre-membrane is a glycoprotein precursor processed to membrane via cleavage, and the envelope protein plays multiple biological roles, such as virion assembly, receptor binding, and membrane fusion [4]. NS1 and NS2A function in viral propagation, NS3 acts as a proteinase, and NS5, the largest non-structural protein, is a viral RNA polymerase. The three small proteins, NS2B, NS4A, and NS4B, are membrane components of the viral replication complex [4].

Five genotypes of JEV have been identified to date, classified as I, II, III, IV, and V based on the E gene [5]. JEV is prevalent in Asia and parts of Australia. Genotype I (GI) is predominantly found in East and Southeast Asia; genotype II (GII) in Australia and Papua New Guinea; genotype III (GIII) throughout Asia; and genotype IV (GIV) in Indonesia, while genotype V (GV) was originally detected in Muar, Malaysia in 1952 [4,6]. GI and GIII are the dominant genotypes in Korea, while GV began to emerge in Korea in 2010 [7,8].

Among the 24 countries with endemic Japanese encephalitis virus transmission, more than 50,000 cases and over 15,000 associated deaths are reported on an annual basis [9]. Among the patient populations with Japanese encephalitis, those under 14 years of age account for 71% of the total infections [9]. Most patients are either asymptomatic, or the disease manifests as flu-like symptoms [10,11]. In the case of symptomatic patients, headache and fever are common at 2-to-4 days after infection, gradually followed by abdominal pain accompanied by nausea and vomiting. In severe cases, epilepsy and seizures may develop. About 5–20% patients experience acute flaccid paralysis, and >50% have reported mobility impairment. However, no specific remedies for JE have yet to be established [12]. In view of the lack of available antiviral treatments, prevention of the disease is crucial. The World Health Organization (WHO) recommends a widespread JEV immunization program via vaccine in vulnerable countries, and for individuals travelling or relocating to JE-endemic nations [13]. Four different types of vaccines have been developed: mouse brain-derived, inactivated Vero cell culture, SA14-14-2 live attenuated, and live attenuated chimeric vaccines. The currently marketed vaccines are based on genotype III [13]. However, recent studies have shown that genotype III-based vaccines are less effective against JEV GV, highlighting concerns about reinfection and new infections in the vaccinated population [14].

Conventional JEV diagnostic methods require blood or cerebrospinal fluid (CSF) to extract viruses or antibodies, which are detected with a range of methods, such as reverse transcription–polymerase chain reaction (RT-PCR), immunofluorescence assay (IFA), hemagglutination assay, and IgM antibody capture enzyme-linked immunosorbent assay (MAC ELISA) [15]. However, these procedures are time-consuming, and their application is a challenge in low- and middle-income countries (LMIC) owing to high cost and technical issues. Earlier findings additionally indicate that a MAC–ELISA-incorporated serum has a problem of cross-reactivity with other *Flaviviruses*, in particular, dengue virus [16,17].

Various diagnostic technologies have been developed in recent years, one of which is the DNA endonuclease-targeted CRISPR trans reporter (DETECTR) that uses isothermal amplification with clustered regularly interspaced short palindromic repeats (CRISPR), along with the CRISPR-associated system (Cas) [18]. CRISPR-Cas is one of the adaptive immune systems of bacteria and archaea. In particular, Cas12a from *Lachnospiraceae* bacterium (LbaCas12a) has *trans*-cleavage activity whereby it detects a complementary sequence to guide RNA (gRNA) next to the protospacer-adjacent motif (PAM) [19]. To amplify nucleic acids, DETECTR uses recombinase polymerase amplification (RPA), which is an alternative isothermal amplification method to PCR [20]. Detection is conducted through various methods, such as fluorescence assays and lateral flow assays (LFA). Using DETECTR, nucleic acids of viruses, such as SARS-CoV-2, influenza virus, and SFTSV, can be effectively detected at the laboratory level [21,22,23,24]. In the current study, JEV GI, GIII, and GV nucleic acids were successfully detected using the fluorescence assay and LFA via DETECTR in the laboratory. We further designed a novel one-pot DETECTR (OP DETECTR) method and compared its utility with that of conventional DETECTR.

## 2. Materials and Methods

### 2.1. Cells and Viruses

BHK-21 cells (Korean Cell Line Bank, Seoul, Republic of Korea) were grown in Dulbecco’s modified Eagle’s medium (DMEM) (Hyclone, Logan, UT, USA) with 10% fetal bovine serum (FBS) (Capricorn, Ebsdorfergrund, Germany) and 1% penicillin and streptomycin (P/S) (Genomicbase, Seoul, Republic of Korea). The Japanese encephalitis virus genotype I (JEV GI) (NCCP43133, National Culture Collection for Pathogens, Cheongju, Republic of Korea) was propagated in BHK-21 cells at passage 20, with a change in medium to DMEM with 2% FBS and 1% P/S. Virus titers were determined in 6-well culture plates. Influenza A virus (IAV) (A/Puerto Rico/8/1934), influenza B virus (IBV) (B/Brisbane/60/2008), hepatitis C virus (HCV) (JFH-1), and hepatitis E virus (HEV) (47832c) were propagated and tittered as described previously [25,26,27,28]. All virus experiments were performed at biosafety level-2 (BSL-2).

### 2.2. Synthesis of RNA via In Vitro Transcription

JEV GI RNA extraction was performed using the AccuPrep^®^ Viral RNA Extraction Kit (Bioneer, Daejeon, Republic of Korea). Complementary DNA (cDNA) was synthesized using the TOPscript™ RT-PCR Kit (Enzynomics, Daejeon, Republic of Korea) with a random hexamer. JEV GIII NS4B (GenBank: EF623989.1, nucleotides 6912 to 7649) and GV NS1 genes (GenBank: JF915894.1, nucleotides 2481 to 3542) were synthesized by Macrogen (Seoul, Republic of Korea). PCR of JEV GI was conducted using the prepared cDNAs and PCR of GIII and GV performed using the synthesized plasmid gene. Primers containing the T7 promoter sequence and Phusion^®^ High-Fidelity PCR Master Mix with HF Buffer (New England Biolabs, MA, USA; Table S1) were employed. JEV RNAs were synthesized via IVT using PCR products and the mMESSAGE mMACHINE T7 Transcription kit (Invitrogen, MA, USA). Synthesized RNAs were purified using a GeneJET RNA Cleanup and Concentration Micro Kit (Thermo Scientific™, Waltham, MA, USA).

### 2.3. Heating Unextracted Diagnostic Samples to Obliterate Nucleases (HUDSON)

For viral RNA extraction from JEV GI, IAV, IBV, HCV, and HEV culture media, HUDSON was carried out as described previously [28]. Tris(2-carboxyethyl)phosphine hydrochloride (TCEP) and ethylenediaminetetraacetic acid (EDTA) were added to virus samples at concentrations of 100 mM and 1 mM, followed by incubation at 50 °C for 20 min and 95 °C for 5 min. Samples were aliquoted into1.5 mL tubes and stored at −80 °C.

### 2.4. Designing Primers for RPA and Guide RNAs for DETECTR

For RPA and DETECTR, we designed the primers and gRNAs based on NCBI database. Primers and gRNA for JEV GI were designed based on nucleotide sequences of JEV strain K05GS (GenBank: KR908702.1), those for JEV GIII based on JEV isolate 04940-4 (GenBank: EF623989.1), and those for JEV GV based on JEV strain XZ0934 (GenBank: JF915894.1).

**Figure 1:**
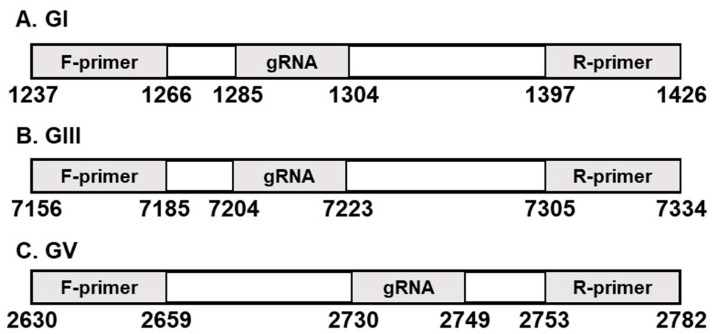
Location of RT-RPA primers and gRNAs specific for JEV genotypes. (**A**) Genotype I; (**B**) Genotype III; (**C**) Genotype V.

Primers for RT-RPA were designed in keeping with the TwistAmp^®^ DNA Amplification Kit Assay manual (Table 1). Strain-specific RPA primer sets were designed for viral RNA amplification with RT-RPA. For JEV GI, the sequence of the forward primer corresponded to nucleotides 1237 to 1266, and that of the reverse primer to nucleotides 1397 to 1426. For JEV GIII, the sequence of the forward primer corresponded to nucleotides 7156 to 7185, and that of reverse primer to nucleotides 7305 to 7334. For JEV GV, the sequence of the forward primer corresponded to nucleotides 2630 to 2659, and that of reverse primer to nucleotides 2753 to 2782 (Figure 1). All reverse primers were in the reverse complementary orientation. The gRNA and RPA primers were aligned with JEV genotypes (Figures S1 and S2).

JEV gRNA was designed in accordance with the PAM sequence (TTTV, V is a A, G, or C) based on sequences identified earlier in the NCBI. The gRNAs of JEV GI were based on nucleotide positions 1285 to 1304, JEV GIII based on nucleotides 7204 to 7223, and JEV GV based on nucleotides 2730 to 2749 (Bioneer, Daejeon, Republic of Korea; Figure 1, Table 2).

### 2.5. DETECTR

RT-RPA was conducted with the TwistAmp^®^ Basic Kit (TwistDx, Cambridge, UK) for viral RNA amplification. RT-RPA products were generated with a reaction containing rehydration buffer (29.5 µL), 10 µM of forward and reverse primers (2.4 µL), 1 μL of SuperScript IV Reverse Transcriptase (Invitrogen, Waltham, MA, USA), 1 µL of RNase inhibitor (Enzynomics, Republic of Korea), and 2.5 µL of 280 mM magnesium acetate following the manufacturer’s instructions. Samples and nuclease-free water were added to the complex up to a final volume of 50 µL before adding magnesium acetate. In this study, the sample volume used was 5 µL. RNAs were added to the RPA complex (1 × 10^3^ to 1 × 10^0^ RNA copies) with incubation at 42 °C for 40 min. To compare the sequences of primer sets between genotypes, primer sequences were aligned according to JEV genotypes (Figure S1). The same concentration of reagents was applied to detect each JEV genotype.

LbaCas12a *trans*-cleavage assays were conducted in keeping with previous experiments [23]. To generate a reaction complex, EnGen^®^ Lba Cas12a (Cpf1, LbaCas12a) (New England Biolabs, Ipswich, MA, USA) and gRNA were added to 1 × NEB 2.1 buffer to final concentrations of 50 nM and 62.5 nM, respectively. The complexes generated were incubated at 37 °C for 30 min. After preparation of RT-RPA products and LbaCas12a-gRNA complex, 1 × NEB 2.1 buffer (80 µL), LbaCas12a-gRNA complex (18 µL), RPA products (2 µL), and 10 µM FQ-labeled reporter (2 µL) (/56-FAM/TTATT/3IABkFQ/; Integrated DNA Technologies, Coralville, IA, USA) were added to each well of 96-well microplates. Fluorescence assays were conducted at 0 and 10 min, and 96-well microplates were incubated at 37 °C before obtaining measurements at 10 min. To compare gRNA sequences between genotypes, sequences were aligned according to JEV genotypes (Figure S2).

LFA used the same LbaCas12a-gRNA complex of DETECTR. Complexes for LFA were generated in a 1.5 mL tube with 38 µL of 1 × NEB 2.1 buffer, 36 µL of LbaCas12a-gRNA complex, 2 µL of 10 µM lateral flow cleavage reporter (/56-FAM/TTATT/3Bio/; Integrated DNA Technologies, Coralville, IA, USA) and 4 µL RPA products and incubated at 37 °C for 10 min. Next, Milenia HybriDetect 1 lateral flow strips (Milenia, Giesesen, Germany) were dipped in the sample tubes and data analyzed after 2 min.

### 2.6. One-Pot DETECTR (OP DETECTR)

**Figure 2:**
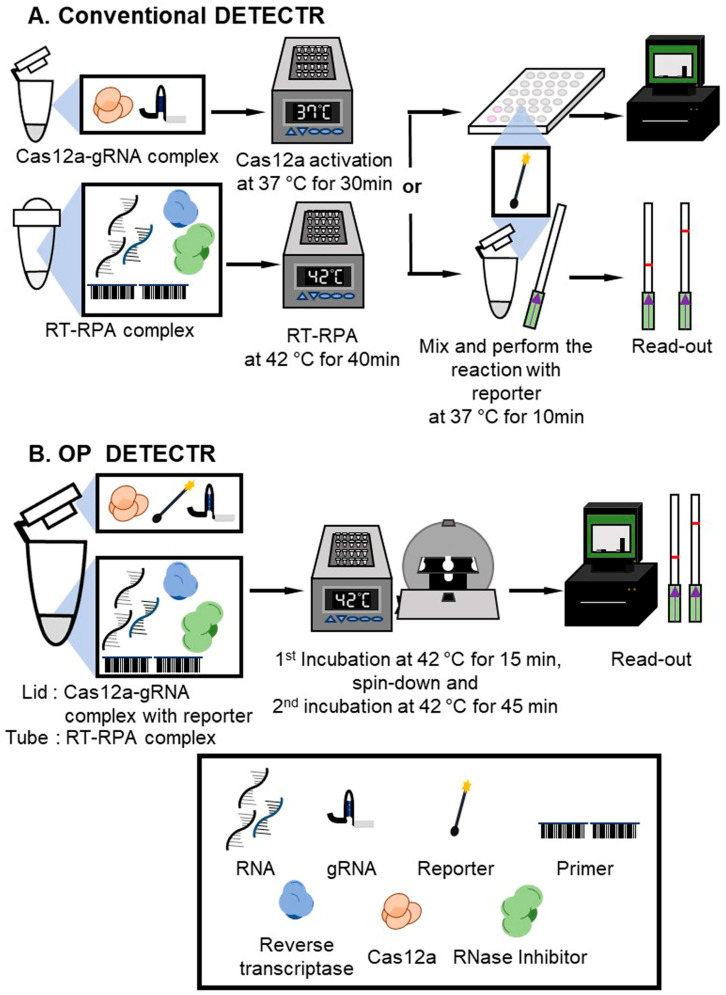
Schematic diagrams of DETECTR and OP DETECTR workflows. (**A**) Conventional DETECTR; (**B**) OP DETECTR.

One-pot DETECTR (OP DETECTR) is a simplified process involving incubation of both RT-RPA and LbaCas12a-gRNA complexes in the same tube at the same time and temperature (Figure 2B). The OP DETECTR process was designed in keeping with previous reports [29,30]. The method comprised two components: A, with the RT-RPA reaction proceeding within the tube, and B, consisting of the LbaCas12a-gRNA complex and reporter for activation in the lid. Component A comprised rehydration buffer (29.5 µL), 10 µM of forward primer (2.4 µL), 10 µM of reverse primer (2.4 µL), reverse transcriptase (1 µL) and RNase inhibitor (1 µL), which was divided into two new tubes and RNA, with nuclease-free water added to a volume of 27.25 µL. RPA powder was resuspended in 13 µL of nuclease-free water and divided into 6.5 µL aliquots, followed by the addition of 1.25 µL of 280 mM magnesium acetate into each tube. Component B comprised 10 × NEB 2.1 buffer (2.5 µL), 1 µM of Cas12a (1 µL), 1 µM of gRNA (0.5 µL), and 10 µM of FQ-labeled reporter (1 µL), which was placed in the lid of tubes and closed carefully to ensure no spillage of the contents. The same concentration of reagents was applied to detect each JEV genotype.

Tubes were incubated at 42 °C for 15 min, briefly spun to ensure mixing of Component B from the lid into the tubes, and further incubated at 42 °C for 45 min. Results from real-time RT-RPA were evaluated using an Axxin T16-ISO instrument (Axxin, Fairfield, Australia).

For LFA detection, Component A was the same as that used for real-time RT-RPA, while Component B was composed of 1 × NEB 2.1 buffer (29 µL), 1 µM of Cas12a (8 µL), 1 µM of gRNA (1 µL), and 10 µM of lateral flow cleavage reporter (1 µL). The process was identical to that described for Figure 2B. After 45 min, Milenia HybriDetect 1 lateral flow strips were dipped in the reaction tubes, and results were evaluated after 2 min.

## 3. Results

### 3.1. Design of JEV DETECTR

We aimed to develop a CRISPR-Cas12a-based detection system for JEV with minimal equipment and a maximal reduction of overall assay time. Viral RNAs were amplified using reverse transcription-recombinase polymerase amplification (RT-RPA). Amplified products were treated with an LbaCas12a-gRNA complex and ssDNA-fluorophore quencher (FQ)-labeled reporter for the fluorescence assay or ssDNA-FAM-Biotin (FB) reporter for LFA (Figure 2A). With an effort to further simplifying the process, OP DETECTR was designed to allow for the performance of DETECTR within a single tube, with RT-RPA and the incubation with the LbaCas12a-gRNA complex conducted separately (Figure 2B).

### 3.2. Specificity of JEV DETECTR

**Figure 3:**
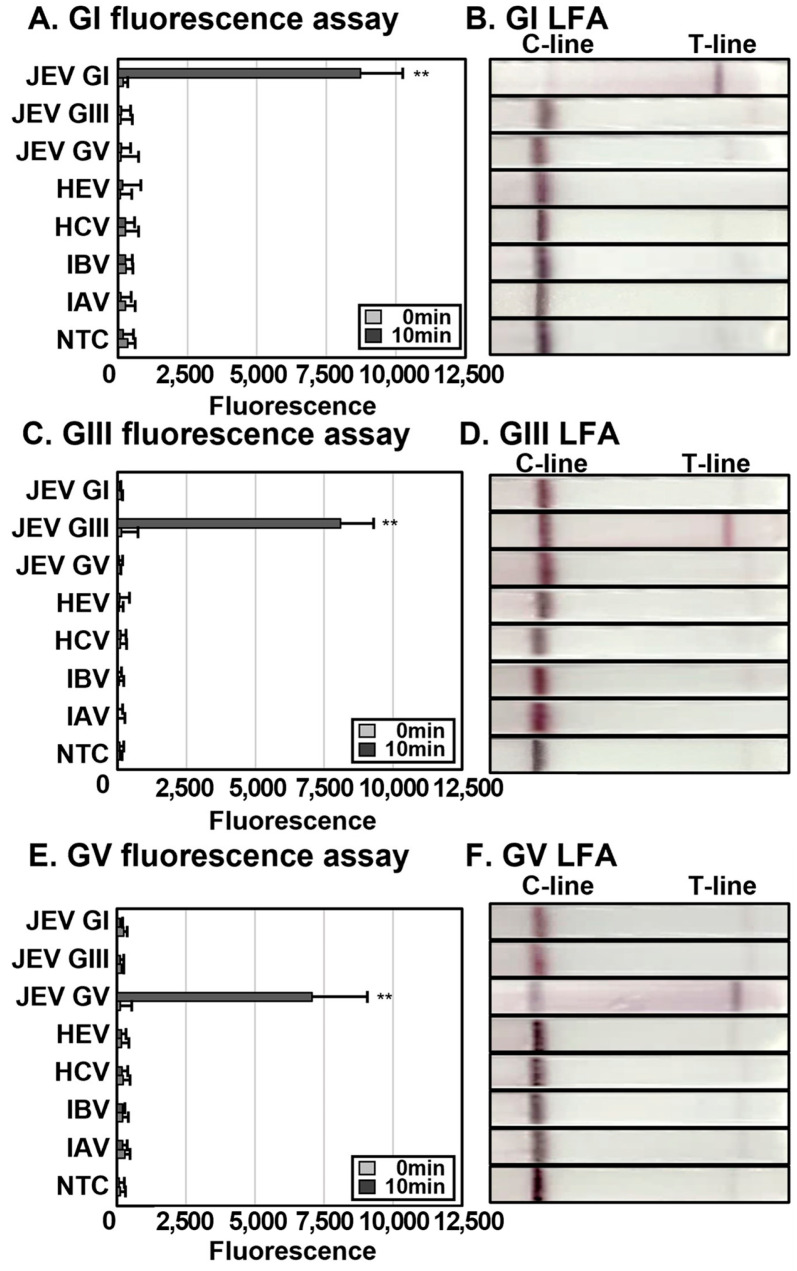
Establishment of JEV DETECTR specificity according to virus type. RNAs (1 × 10^3^ PFU per reaction of JEV GI, IAV and IBV, 1 × 10^3^ FFU per reaction of HCV and HEV) were extracted via HUDSON and amplified using RT-RPA. RNAs of JEV GIII and GV were synthesized via IVT and 1 × 10^3^ RNA copies per reaction used for RT-RPA. RNAs were amplified with primer sets corresponding to each genotype. For the *trans*-cleavage assay, gRNAs specific for each genotype were used. The results of DETECTR of RT-RPA products for each JEV gRNA were detected via fluorescence assay and LFA. Fluorescence was measured after reaction of RT-RPA amplicons with the LbaCas12a-gRNA complex at 37 °C for 10 min (**A**,**C**,**E**). Results of LFA were assessed at 2 min after inserting the strip into the sample (**B**,**D**,**F**). Error bars represent mean ± s.d. (*n* = 3 replicates). The asterisk (**) denotes a significant difference between 0-min and 10-min samples determined based on *p*-values of a two-sample *t*-test (*p* < 0.05). C-line, control line; T-line, test line; NTC, non-template control.

To determine the specificity of JEV gRNAs, IAV, IBV, HCV, and HEV were used as controls, and viral RNAs extracted with HUDSON. The titers of IAV, IBV, and JEV GI were 1 × 10^3^ (plaque forming units (PFUs) per mL (equivalent to 5.01 × 10^4^, 3.46 × 10^7^ and 2.88 × 10^7^ RNA copies/mL, respectively), and those of HCV and HEV were 1 × 10^3^ fluorescence-forming units (FFUs) per mL (equivalent to 1.02 × 10^7^, and 1.5 × 10^7^ RNA copies/mL, respectively). RNAs of GIII and GV were synthesized via in vitro transcription (IVT). RNAs were added at 1 × 10^3^ RNA copies per reaction for RT-RPA. In both fluorescence assay and LFA, no cross-reactivity of gRNAs with other viruses was observed, and gRNAs specific for the JEV genotype solely reacted with designated JEV RT-RPA amplicons (Figure 3).

### 3.3. Determination of Limit of Detection (LoD) of Conventional JEV DETECTR

**Figure 4:**
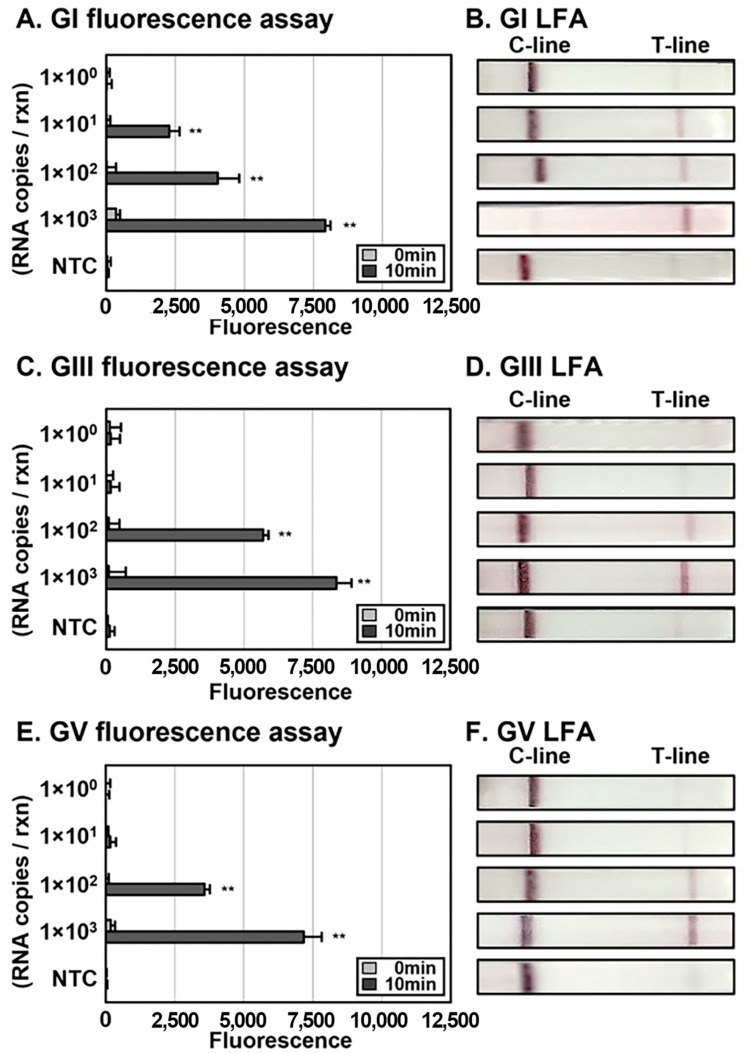
Detection of JEV DETECTR sensitivity for each genotype. RNAs were added to RT-RPA products (1.0 × 10^3^ to 1.0 × 10^0^ RNA copies per reaction) and used to amplify viral nucleic acids via RT-RPA with primer sets specific for each genotype. The results of DETECTR were detected using both fluorescence assays and LFA. Levels of fluorescence were measured after reacting RT-RPA amplicons with the LbaCas12a-gRNA complex at 37 °C for 10 min (**A**,**C**,**E**). LFA results were assessed at 2 min after inserting the strip into the sample (**B**,**D**,**F**). Error bars represent mean ± s.d., (*n* = 3 replicates). The asterisk (**) denotes a significant difference between 0 min and 10 min, determined based on the *p*-value of a three-sample *t*-test (*p* < 0.05). C-line, control line; T-line, test line; NTC, non-template control.

To establish the sensitivity of DETECTR, we synthesized RNAs of the three JEV genotypes using IVT, which were used for RT-RPA. RNAs were serially 10-fold diluted with RNase-free distilled water and added to the RT-RPA complex (1.0 × 10^3^ to 1.0 × 10^0^ copies per reaction). JEV GI DETECTR effectively detected a minimum of 1 × 10^1^ copies per reaction in both fluorescence assays and LFA (Figure 4A,B). However, for JEV GIII and GV, LoD of DETECTR was 1 × 10^2^ copies per reaction in both assays (Figure 4C–F). The LoD of the RT-PCR-based detection of in vitro-transcribed JEV GI, GIII, and GV RNAs was 1 × 10^2^ RNA copies per reaction, while that of DETECTR was 10 copies per reaction for GI and 1 × 10^2^ copies per reaction for GIII and GV [31].

### 3.4. Determination of LoD of OP JEV DETECTR

**Figure 5:**
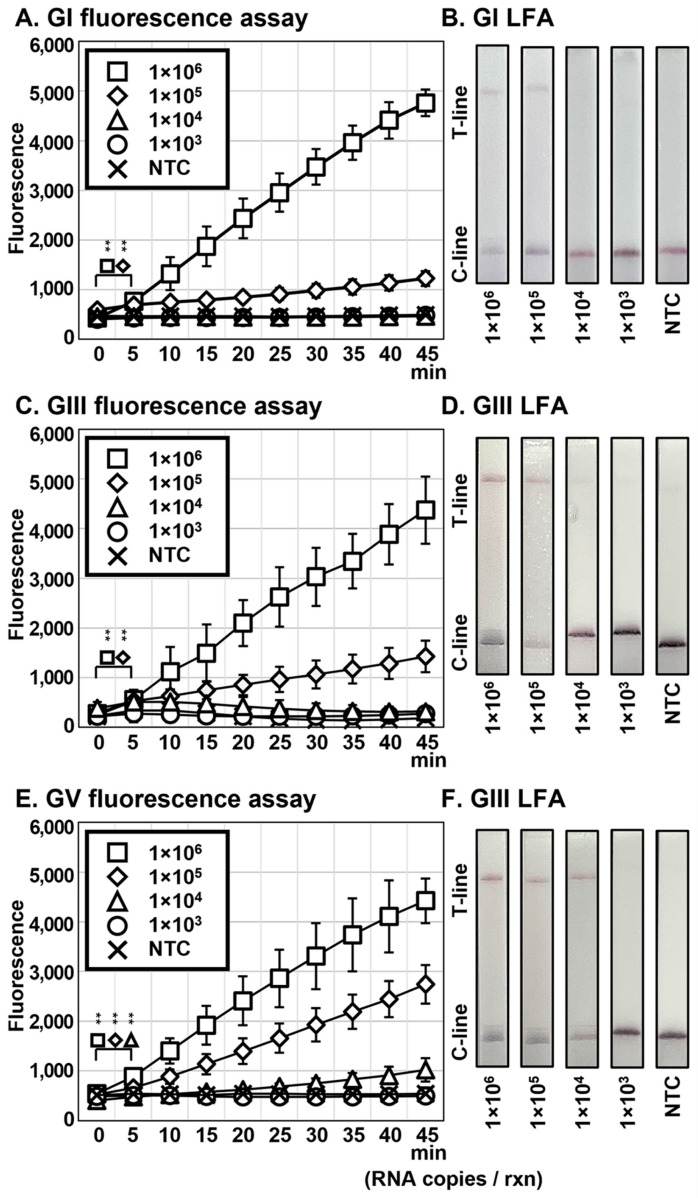
Detection of OP DETECTR sensitivity for each genotype. The RNAs were added to OP DETECTR from 1.0 × 10^6^ to 1.0 × 10^3^ RNA copies per reaction. The fluorescence assays were detected in real time by Axxin T16-ISO (**A**,**C**,**E**), and LFAs were detected 2 min after the LFA strips were placed in the tubes (**B**,**D**,**F**). The sensitivity was identical in both assays. Error bars represent mean ± s.d., where *n* = 3 replicates. The asterisk (**) on square, diamonds, and triangle forms denotes significant difference of 1.0 × 10^6^, 1.0 × 10^5^, and 1.0 × 10^4^ RNA copies between 0 min and samples, respectively, which were determined by the *p*-value of a three-sample *t*-test (*p* < 0.05). C-line, control line; T-line, test line; NTC, non-template control.

Conventional DETECTR uses two different temperatures, specifically, 42 °C for RT-RPA and 37 °C for incubation of the LbCas12a-gRNA complex. In addition, RT-RPA is prone to contamination during the process of transferring the contents in the tubes. Here, we further developed one-pot (OP) DETECTR to reduce equipment requirements and contamination using the method shown in Figure 2B. RNAs were added to the complex from 1.0 × 10^6^ to 1.0 × 10^3^ copies per reaction and fluorescence of OP DETECTR detected in real time with the Axxin T16-ISO instrument. The procedure detected a minimum of 1 × 10^5^ copies per reaction of JEV GI and GIII and 1 × 10^4^ copies per reaction of JEV GV in both fluorescence assays and LFA (Figure 5). Overall, the sensitivity of OP JEV DETECTR for GI, GIII, and GV was 10^4^-, 10^2^-, and 10^1^-fold lower than the conventional technique, respectively.

## 4. Discussion

Five genotypes of JEV are widely distributed in Asia and Australia [12]. Although all genotypes are prevalent in Asia, GI and GIII are mainly located in temperate climates, while GII and GIV are endemic in tropical climates [6]. A recent study demonstrated low efficiency of conventional vaccines for GV, with a re-infection of some patients vaccinated against JE [7,14,32]. GI, GIII, and GV were selected for detection in the current study, since GI and GIII are more widely distributed than other strains and GV has increased in frequency in recent years [6,7].

Various diagnostic methods have been explored for JEV in terms of sensitivity, specificity, and speed. Additionally, conventional diagnostic methods have been comprehensively evaluated with the aim of improving their shortcomings. In the case of RT-PCR, a commonly used method of nucleic acid-based diagnosis, attempts have been made to enhance its sensitivity and shorten the running time [33,34]. Likewise, experiments to reduce the time taken for ELISA have been attempted, especially in the case of MAC–ELISA, which has also been further evaluated to eliminate false-positive results [16,17,35]. Data from this investigation showed that results with DETECTR could be obtained over a relatively short time with high specificity and sensitivity. The sensitivity of RT-PCR-based detection of in vitro-transcribed JEV GI, GIII, and GV RNAs was down to 1 × 10^2^ RNA copies per reaction [31]. LoD of DETECTR for JEV GI was 10 RNA copies per reaction, while that for GIII and GV was 1 × 10^2^ copies per reaction. Compared with RT-PCR-based diagnosis, JEV DETECTR was sufficiently sensitive with no significant differences for GIII and GV but 10-fold more sensitive for GI (Figure 4). In this respect, DETECTR may effectively serve as a novel diagnostic tool for detection of JEV.

Detection of various types of viral nucleic acids, such as SARS-CoV-2, influenza A virus, influenza B virus, and SFTSV using DETECTR has been examined [21,22,23,24]. DETECTR was able to effectively detect 1 × 10^1^ viral RNA copies per reaction of JEV GI in both fluorescence assays and LFA, which was more sensitive relative to the detection of GIII and GV (Figure 4). However, further investigations with clinical samples, such as patient CSF, are essential to confirm the diagnostic efficacy of DETECTR. In particular, it is necessary to establish whether data obtained with HUDSON, which is used to simplify the process, are consistent with the use of conventional JEV diagnostic samples. In addition, extracellular RNAs in CSF can be utilized as a positive control for RPA and DETECTR [24,36].

LFA provides a low-cost, simple, rapid, and portable detection method [37]. Since the main outbreak and endemic areas of JEV are Southeast Asian low- and middle-income countries (LMIC) with a relatively underdeveloped medical infrastructure, LFA provides a simple and inexpensive diagnostic tool requiring only isothermal devices (such as a heat block). Accordingly, LFA was selected as the detection method in the current study. To optimize the developability, we compared the sensitivity and specificity of LFA with that of fluorescence assays. Although the drawback began to appear faintly on the test line of the non-template control (NTC) after 2 min, LoD and specificity of LFA were similar to those of fluorescence assays (Figure 3). Given that the sensitivity of LFA is comparable to that of the fluorescence assay, if the buffer for LFA is prepared in the form of a master mix, it will be sufficient as a field diagnosis technology in the presence of only heat blocks. The low cost and simplicity of the method supports its utility as a diagnostic tool, not only in JEV-endemic LMIC but also military bases, rural and medically underdeveloped regions.

To facilitate more rapid and simple diagnosis, we designed one-pot (OP) DETECTR based on previous studies [29,30]. To allow RT-RPA and LbaCas12a-gRNA complexes to react separately, each complex was aliquoted into a tube and lid, respectively. While significant differences were observed 5 min after the reaction, results of both the fluorescence assay and LFA were less sensitive compared to conventional DETECTR (Figure 5). The maximum difference between OP DETECTR and conventional DETECTR was 10^4^-fold for JEV GI, and a minimum difference was 10^1^-fold for GV. For JEV GIII, the sensitivity of OP DETECTR was 10^2^-fold lower than conventional DETECTR. We propose two reasons to explain this difference in LoD between the methods. Firstly, the amplification time using RT-RPA is shorter for OP-DETECTR compared to the conventional technique. For sufficient amplification, conventional DETECTR requires more than 30 min for RT-RPA, while the corresponding time for OP DETECTR is 15 min. Accordingly, we infer that this time is sufficient for RT-RPA to proceed effectively in cases of high RNA copies but not low RNA copies. In addition, although RT-RPA proceeds before spin down, it is possible that the LbaCas12a-gRNA complex, which is added afterwards, inhibits the RT-RPA reaction to an extent. Another potential reason is that the temperature of incubation of the LbaCas12a-gRNA complex is higher than that used for the conventional method. In OP DETECTR, the LbaCas12a-gRNA complex was incubated at 42 °C. Meanwhile, the temperature of LbaCas12a activation has been documented as 16–48 °C according to the manual of EnGen^®^ Lba Cas12a (Cpf1), in experiments by Fuchs et al. [38]. The ribonucleoprotein (RNP) complex consisting of LbaCas12a protein loaded with gRNA (crRNA) showed low activity at temperatures over 41.2 °C [38]. Therefore, the higher incubation temperature of the LbaCas12a-gRNA complex may inhibit *trans*-cleavage activity.

In this study, we have achieved sensitive and rapid genotype-specific detection of JEV using DETECTR. The sensitivity of DETECTR was sufficient in addition to reduced working time and cost compared to other conventional methods, such as RT-PCR and ELISA. We further designed OP DETECTR suited for TwistAmp^®^ Basic Kit, which operated well at high RNA copy numbers. Despite its lower sensitivity, OP DETECTR could be used to minimize contamination or in situations where a quick test is required. In summary, DETECTR and OP DETECTR require shorter test times and simple steps and can be effectively utilized as diagnostic tools, even in areas with limited experimental facilities. In view of its collective advantages, DETECTR may therefore serve as an effective alternative to RT-PCR and ELISA in LMICs with financial and technical constraints.

## Figures and Tables

**Table 1:** RPA primers for JEV DETECTR.

Genotype	Primer	Sequence
Genotype I	JEV-GI-F	GACAGCAGCTACGTGTGCAAACAAGGCTTT
	JEV-GI-R	CCGAGGTGGTGGTTCCGTGCACGAATATGC
Genotype III	JEV-GIII-F	TGCCACGAGGCGTGCCTTTTACCGACCTAG
	JEV-GIII-R	TGTCCTTCTCTGGGCAGCCCTGAGTGCTTC
Genotype V	JEV-GV-F	GGAAGGCATTTGTGGAGTGAGATCAGTCAC
	JEV-GV-R	AATGGAGCTGGTCGGTATCTTCCTGATGGT

**Table 2:** gRNAs for JEV DETECTR.

Genotype	gRNA	Sequence	PAM
Genotype I	JEV-GI-gRNA	CCGAAAAGUCCACAUCCAUU	TTTC
Genotype III	JEV-GIII-gRNA	ACCCCAACAGCCAAGGAAGA	TTTG
Genotype V	JEV-GV-gRNA	UCCACCACCACACUAAGAUC	TTTG

## Data Availability

All data and materials supporting the conclusions are described and included in this manuscript.

## Supplementary Materials

The following supporting information can be downloaded at: https://www.mdpi.com/article/10.3390/bios13080769/s1, Table S1: Primers for in vitro transcription; Figure S1: Alignment of RT-RPA primers with target sequences of JEV genotypes; Figure S2: Alignment of gRNAs with target sequences of JEV genotypes.

## References

[1] Park S.L., Huang Y.-J.S., Vanlandingham D.L. (2022). Re-Examining the Importance of Pigs in the Transmission of Japanese Encephalitis Virus. Pathogens.

[2] Miller R.H., Masuoka P., Klein T.A., Kim H.-C., Somer T., Grieco J. (2012). Ecological Niche Modeling to Estimate the Distribution of Japanese Encephalitis Virus in Asia. PLoS Neglected Trop. Dis..

[3] Sumiyoshi H., Mori C., Fuke I., Morita K., Kuhara S., Kondou J., Kikuchi Y., Nagamatu H., Igarashi A. (1987). Complete nucleotide sequence of the Japanese encephalitis virus genome RNA. Virology.

[4] Chambers T.J., Hahn C.S., Galler R., Rice C.M. (1990). Flavivirus genome organization, expression, and replication. Annu. Rev. Microbiol..

[5] Solomon T., Ni H., Beasley D.W.C., Ekkelenkamp M., Cardosa M.J., Barrett A.D.T. (2003). Origin and Evolution of Japanese Encephalitis Virus in Southeast Asia. J. Virol..

[6] Mackenzie J.S., Williams D.T., Smith D.W. (2006). Japanese Encephalitis Virus: The Geographic Distribution, Incidence, and Spread of a Virus with a Propensity to Emerge in New Areas. Perspect. Med. Virol..

[7] Lee A.-R., Song J.M., Seo S.-U. (2022). Emerging Japanese Encephalitis Virus Genotype V in Republic of Korea. J. Microbiol. Biotechnol..

[8] Yun S.-M., Cho J.E., Ju Y.-R., Kim S.Y., Ryou J., Han M.G., Choi W.-Y., Jeong Y.E. (2010). Molecular epidemiology of Japanese encephalitis virus circulating in South Korea, 1983–2005. Virol. J..

[9] World Health Organization (2006). Japanese encephalitis vaccines. Wkly. Epidemiol. Rec. Relev. Épidémiol. Hebd..

[10] Campbell G.L., Hills S.L., Fischer M., Jacobson J.A., Hoke C.H., Hombach J.M., Ginsburg A.S. (2011). Estimated global incidence of Japanese encephalitis: A systematic review. Bull. World Health Organ..

[11] Solomon T., Winter P.M. (2004). Neurovirulence and host factors in flavivirus encephalitis—Evidence from clinical epidemiology. Emergence and Control of Zoonotic Viral Encephalitides.

[12] Misra U.K., Kalita J. (2010). Overview: Japanese encephalitis. Prog. Neurobiol..

[13] Yun S.-I., Lee Y.-M. (2014). Japanese encephalitis: The virus and vaccines. Hum. Vaccines Immunother..

[14] Cao L., Fu S., Gao X., Li M., Cui S., Li X., Cao Y., Lei W., Lu Z., He Y. (2016). Low Protective Efficacy of the Current Japanese Encephalitis Vaccine against the Emerging Genotype 5 Japanese Encephalitis Virus. PLoS Neglected Trop. Dis..

[15] Bharucha T., Shearer F.M., Vongsouvath M., Mayxay M., de Lamballerie X., Newton P.N., Dubot-Pérès A. (2020). A need to raise the bar—A systematic review of temporal trends in diagnostics for Japanese encephalitis virus infection, and perspectives for future research. Int. J. Infect. Dis..

[16] Jacobson J.A., Hills S.L., Winkler J.L., Mammen M., Thaisomboonsuk B., Marfin A.A., Gibbons R.V. (2007). Evaluation of three immunoglobulin M antibody capture enzyme-linked immunosorbent assays for diagnosis of Japanese encephalitis. Am. J. Trop. Med. Hyg..

[17] Martin D.A., Biggerstaff B.J., Allen B., Johnson A.J., Lanciotti R.S., Roehrig J.T. (2002). Use of Immunoglobulin M Cross-Reactions in Differential Diagnosis of Human Flaviviral Encephalitis Infections in the United States. Clin. Vaccine Immunol..

[18] Mustafa M.I., Makhawi A.M. (2021). SHERLOCK and DETECTR: CRISPR-Cas Systems as Potential Rapid Diagnostic Tools for Emerging Infectious Diseases. J. Clin. Microbiol..

[19] Li S.-Y., Cheng Q.-X., Wang J.-M., Li X.-Y., Zhang Z.-L., Gao S., Cao R.-B., Zhao G.-P., Wang J. (2018). CRISPR-Cas12a-assisted nucleic acid detection. Cell Discov..

[20] Daher R.K., Stewart G., Boissinot M., Bergeron M.G. (2016). Recombinase Polymerase Amplification for Diagnostic Applications. Clin. Chem..

[21] Park B.J., Yoo J.R., Heo S.T., Kim M., Lee K.H., Song Y.-J. (2022). A CRISPR-Cas12a-based diagnostic method for multiple genotypes of severe fever with thrombocytopenia syndrome virus. PLoS Neglected Trop. Dis..

[22] Sun Y., Yu L., Liu C., Ye S., Chen W., Li D., Huang W. (2021). One-tube SARS-CoV-2 detection platform based on RT-RPA and CRISPR/Cas12a. J. Transl. Med..

[23] Park B.J., Park M.S., Lee J.M., Song Y.J. (2021). Specific Detection of Influenza A and B Viruses by CRISPR-Cas12a-Based Assay. Biosensors.

[24] Broughton J.P., Deng X., Yu G., Fasching C.L., Servellita V., Singh J., Chiu C.Y. (2020). CRISPR–Cas12-based detection of SARS-CoV-2. Nat. Biotechnol..

[25] Schemmerer M., Apelt S., Trojnar E., Ulrich R.G., Wenzel J.J., Johne R. (2016). Enhanced Replication of Hepatitis E Virus Strain 47832c in an A549-Derived Subclonal Cell Line. Viruses.

[26] Yi M. (2010). Hepatitis C virus: Propagation, quantification, and storage. Curr. Protoc. Microbiol..

[27] Kim J.I., Lee S., Lee G.Y., Park S., Bae J.-Y., Heo J., Kim H.-Y., Woo S.-H., Lee H.U., Ahn C.A. (2019). Novel Small Molecule Targeting the Hemagglutinin Stalk of Influenza Viruses. J. Virol..

[28] Park G., Parveen A., Kim J.-E., Cho K.H., Kim S.Y., Park B.J., Song Y.-J. (2019). Spicatoside A derived from *Liriope platyphylla* root ethanol extract inhibits hepatitis E virus genotype 3 replication in vitro. Sci. Rep..

[29] Li Y., Shi Z., Hu A., Cui J., Yang K., Liu Y., Deng G., Zhu C., Zhu L. (2022). Rapid One-Tube RPA-CRISPR/Cas12 Detection Platform for Methicillin-Resistant *Staphylococcus aureus*. Diagnostics.

[30] Aman R., Mahas A., Marsic T., Hassan N., Mahfouz M.M. (2020). Efficient, rapid, and sensitive detection of plant RNA viruses with one-pot RT-RPA–CRISPR/Cas12a assay. Front. Microbiol..

[31] Nan S., Fan L.I., Kai N.I.E., Fu S.H., Zhang W.J., Ying H.E., Wang H.Y. (2018). TaqMan real-time RT-PCR assay for detecting and differentiating Japanese encephalitis virus. Biomed. Environ. Sci..

[32] Woo J.H., Jeong Y.E., Jo J.E., Shim S.-M., Ryou J., Kim K.-C., Lee W.J., Lee J.-Y. (2020). Genetic Characterization of Japanese Encephalitis Virus Genotype 5 Isolated from Patient, South Korea, 2015. Emerg. Infect. Dis..

[33] Huang J.-L., Lin H.-T., Wang Y.-M., Weng M.-H., Ji D.-D., Kuo M.-D., Liu H.-W., Lin C.-S. (2004). Sensitive and specific detection of strains of Japanese encephalitis virus using a one-step TaqMan RT-PCR technique. J. Med. Virol..

[34] Santhosh S., Parida M., Dash P., Pateriya A., Pattnaik B., Pradhan H., Tripathi N., Ambuj S., Gupta N., Saxena P. (2007). Development and evaluation of SYBR Green I-based one-step real-time RT-PCR assay for detection and quantitation of Japanese encephalitis virus. J. Virol. Methods.

[35] Atchareeya A., Panthuyosri N., Anantapreecha S., Chanama S., Sa-Ngasang A., Sawanpanyalert P., Kurane I. (2008). Cross-reactive IgM responses in patients with dengue or Japanese encephalitis. J. Clin. Virol..

[36] Cavrini F., Della Pepa M.E., Gaibani P., Pierro A.M., Rossini G., Landini M.P., Sambri V. (2011). A rapid and specific real-time RT-PCR assay to identify Usutu virus in human plasma, serum, and cerebrospinal fluid. J. Clin. Virol..

[37] Koczula K.M., Gallotta A. (2016). Lateral flow assays. Essays Biochem..

[38] Fuchs R.T., Curcuru J.L., Mabuchi M., Noireterre A., Weigele P.R., Sun Z., Robb G.B. (2022). Characterization of Cme and Yme thermostable Cas12a orthologs. Commun. Biol..

