# Supplementary material for: A CRISPR-Cas12a-Based Diagnostic Method for Japanese Encephalitis Virus Genotypes I, III, and V
Source: Biosensors (Basel). 2023 Jul 28;13(8):769. doi: 10.3390/bios13080769 (PMC10452572; doi:10.3390/bios13080769)
Supplement: Supplementary file 1 [file biosensors-13-00769-s001.zip › biosensors-2473238-supplementary.pdf]

Supplementary data

A CRISPR-Cas12a-based diagnostic method for Japanese encephalitis virus genotypes I, III, and V

Supplementary data

Table S1. Primers for *in vitro* transcription

Genotype	Primer	Sequence
Genotype I	T7-JEV-GI-F	TAATACGACTCACTATAGGGAGAGACAGCAGCTACGTGTGCAAACAAGGCTTT
	T7-JEV-GI-R	CCGAGGTGGTGGTTCCGTGCACGAATATGCCAACCT
Genotype III	T7-JEV-GIII-F	TAATACGACTCACTATAGGGAGACTGACTGGATTGCCAAGCAT
	T7-JEV-GIII-R	GAATTCCAAACGGCACTGGCTCCATTGTCCC
Genotype V	T7-JEV-GV-F	TAATACGACTCACTATAGGGAGAATGACGTGGAGGCTTG
	T7-JEV-GV-R	GCAAAGAGAATGCTCTTCCCCCATGC

A. JEV GI RPA primer alignment

	1237 … 1266
GI F-primer	5'-GACAGCAGCTACGTGTGCAAACAAGGCTTT-3'
JEV GI	5'-GACAGCAGCTACGTGTGCAAACAAGGCTTT-3'
JEV GIII	5'-GATAGTAGCTATGTGTGCAAACAAGGCTTC-3'
JEV GV	5'-GACAGCAGCTACGTCTGCAAGCAAGGCTAT-3'

	1426 … 1397
GI R-primer	5'-CCGAGGTGGTGGTTCCGTGCACGAATATGC-3'
JEV GI	5'-CCGAGGTGGTGGTTCCGTGCACGAATATGC-3'
JEV GIII	5'-CCGAAGTGGTGGTTCCATGCACAAAAATAC-3'
JEV GV	5'-CTGCTGTTGTGGTTCCATGAACAAACACTC-3'

B. JEV GIII RPA primer alignment

	7156 … 7185
GIII F-primer	5'-TGCCACGAGGCGTGCCTTTTACCGACCTAG-3'
JEV GI	5'-TGCCGCGAGGCGTGCCTTTCACTGACTTGG-3'
JEV GIII	5'-TGCCACGAGGCGTGCCTTTTACCGACCTAG-3'
JEV GV	5'-TTTTGCCAAAAGGCATGCCTTTTACGGATT-3'

	7334 … 7305
GIII R-primer	5'-TGTCCTTCTCTGGGCAGCCCTGAGTGCTTC-3'
JEV GI	5'-TGTTCTTCTCTGAGCAGCTCTGAGTGCCTC-3'
JEV GIII	5'-TGTCCTTCTCTGGGCAGCCCTGAGTGCTTC-3'
JEV GV	5'-CCGTCTCTGAGCTGCCCTCAGGGCTTCAGC-3'

C. JEV GV RPA primer alignment

	2630 … 2659
GV F-primer	5'-GGAAGGCATTTGTGGAGTGAGATCAGTCAC-3'
JEV GI	5'-AGAAGGAGTGTGCGGGGTCAGATCCGTCAC-3'
JEV GIII	5'-GGAAGGAGTGTGCGGAGTCAGATCTGTCAC-3'
JEV GV	5'-GGAAGGCATTTGTGGAGTGAGATCAGTCAC-3'

	2782 … 2753
GV R-primer	5'-AATGGAGCTGGTCGGTATCTTCCTGATGGT-3'
JEV GI	5'-TTGGGGGCTGAGCGATATCTCCCCACGGGC-3'
JEV GIII	5'-TTAGGGGCTGAGCGATATCTTCCCACGGGC-3'
JEV GV	5'-AATGGAGCTGGTCGGTATCTTCCTGATGGT-3'

Figure S1. Alignment of RT-RPA primers with target sequences of JEV genotypes. The nucleic acid sequences of primer sets for one genotype are aligned with those of other genotypes analyzed in this study. All reverse primers are in the reverse-complement orientation.

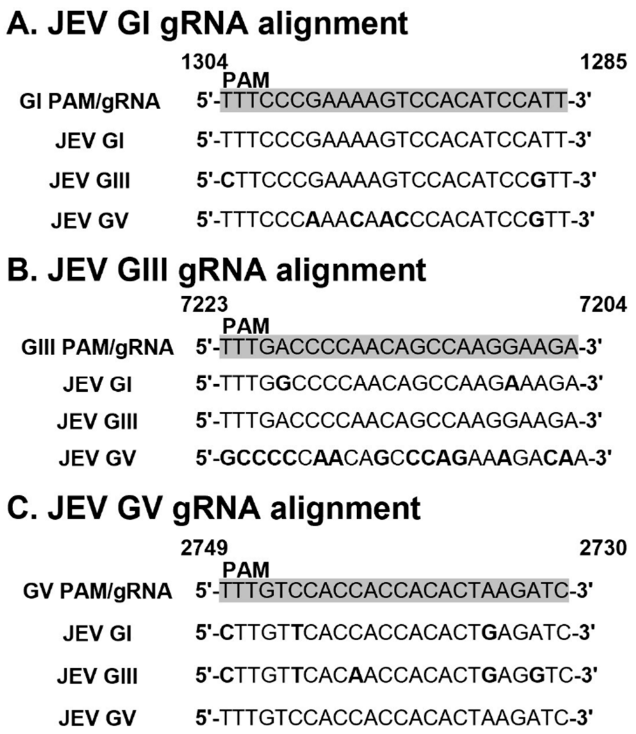

Figure S2. Alignment of gRNAs with target sequences of JEV genotypes. Nucleic acid sequence of gRNA for one genotype is aligned with those of other genotypes analyzed in this study. All gRNAs are in the reverse-complement orientation.
